# Human Papillomavirus Genotypes Distribution in High-Grade Cervical Lesions and Invasive Cervical Carcinoma in Women Living in Mauritania: Implications for Cervical Cancer Prevention and HPV Prophylactic Vaccination

**DOI:** 10.3390/diagnostics14171986

**Published:** 2024-09-08

**Authors:** Mariem Salma Abdoudaim, Mohamed Val Mohamed Abdellahi, Nacer Dine Mohamed Baba, Ralph-Sydney Mboumba Bouassa, Mohamed Lemine Cheikh Brahim Ahmed, Laurent Bélec

**Affiliations:** 1 Unité d’Epidémiologie Moléculaire et Diversité des Microorganismes, Faculté des Sciences et Techniques, Université de Nouakchott, Nouakchott 2373, Mauritanialemine1987@hotmail.fr (M.L.C.B.A.); 2 Laboratoire de Virologie, Hôpital Européen Georges Pompidou, Assistance Publique-Hôpitaux de Paris, 75015 Paris, France; 3 Centre Hospitalier National, Nouakchott 5026, Mauritania; 4 Department of Family Medicine, Faculty of Medicine, Institut du Savoir Montfort, Montfort Hospital, University of Ottawa, Ottawa, ON K1K 0T2, Canada; 5 Ecole Doctorale Régionale (EDR) d’Afrique Centrale en Infectiologie Tropicale, Franceville BP 876, Gabon; 6 Faculté de Médecine Paris Descartes, Université Paris Cité, 75006 Paris, France

**Keywords:** cervical cancer, HPV, HPV detection and genotyping, multiplex real-time PCR, cervical pathology, Mauritania

## Abstract

Cervical cancer related to high-risk human papillomavirus (HR-HPV) is the second female cancer in Mauritania (Northwest Sahelian Africa). We assessed the distribution of HPV genotypes in Mauritanian women with high-grade cervical intraepithelial neoplasia (CIN2/3) or invasive cervical cancer (ICC). A prospective study was conducted in the Centre Hospitalier National, Nouakchott, Mauritania, to collect cervical biopsies among women suspected of CIN2/3 or cancer. HPV DNA detection and genotyping were carried out from formalin-fixed, paraffin-embedded biopsies using multiplex PCR (Human Papillomavirus Genotyping Real-Time PCR Kit, Bioperfectus Technologies Co., Taizhou, China). Fifty biopsies were included from women (mean age: 56.7 years) suffering from CIN2/3 (28.0%) and ICC (72.0%) which corresponded to 32 (64.0%) squamous cell carcinomas (SCC) and 4 (8.0%) adenocarcinomas (ADC). HPV DNA detection was successful in 47 (94.0%) samples. The most prevalent HR-HPV genotypes were HPV-45 (40.4%), HPV-16 (38.3%), HPV-39 and HPV-52 (23.4%), HPV-33 (17.0%), HPV-18 (14.9%), HPV-35 (4.2%), and HPV-56 (2.1%). The majority (93.6%) of HPV-positive biopsies contained at least one HPV type covered by the 9-valent Gardasil-9^®^ vaccine, and 40.9% were infected by multiple vaccine HPV genotypes. To eradicate cervical cancer in Mauritania, prophylactic HPV vaccination must be combined with primary molecular screening of cervical HR-HPV infection.

## 1. Introduction

More than 170 human papillomavirus (HPV) genotypes have been identified [1]. Thirteen types are recognized as high-risk HPV (HR-HPV) and involved in HPV-associated cancers: HPV-16, -18, -31, -33, -35, -39, -45, -51, -52, -56, -58, -59, and -68 [2]. It is widely recognized that persistent HR-HPV infections are causally associated with anogenital cancers (particularly cervical cancer) [3,4,5]. Together, HPV-16 and HPV-18 cause about 70% of cervical cancers worldwide [5,6]. On the other hand, low-risk HPV causes benign pathologies, and HPV-6 and HPV-11 are responsible for about 90% of genital warts worldwide [7].

According to the World Health Organization (WHO), about 660,000 new cases of cervical cancer and 350,000 deaths were reported in 2022, making cervical cancer the ninth deadly female cancer worldwide [8]. Cervical cancer is the second most common cancer for women in developing countries and the second leading cause of cancer-related deaths in sub-Saharan Africa [8,9,10,11,12,13,14]. The estimates for Africa show that 125,699 new cases of cervical cancer (new cancers incidence rate of 18.5% in African women) and 80,614 associated deaths were recorded in 2022 [15]. Cervical cancer-associated mortality in sub-Saharan Africa is up to 10 times higher than in most European countries [9,15,16].

Knowledge of HPV prevalence and HR-HPV genotypes distribution is essential for the implementation of effective prophylactic vaccination programs and appropriate epidemiological monitoring of viral ecology before and after vaccination in specific populations and areas in sub-Saharan Africa [9,17,18,19]. Since the last decade, epidemiological studies have been conducted in several sub-Saharan African countries, showing high heterogeneity in the geographical distribution of the carcinogenic genotypes [9,20,21,22,23,24,25]. For example, adjusted HPV prevalences among sub-Saharan African women were estimated at 33.6%, 19.6%, and 17.4% in Eastern, Western, and Southern Africa, respectively [6].

**Figure 1:**
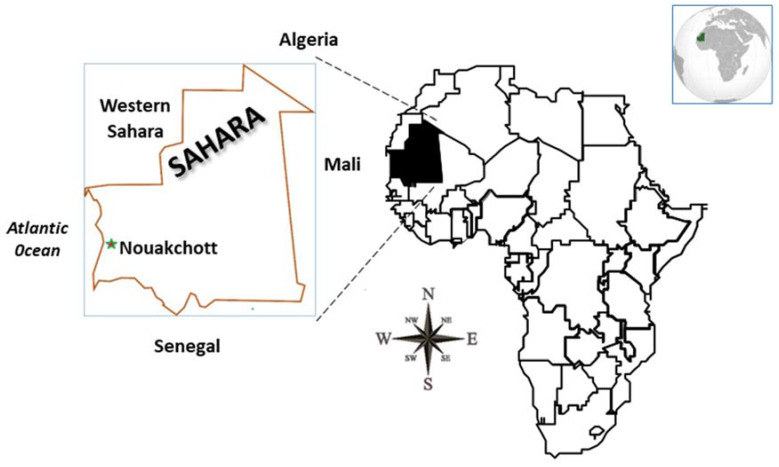
Geographical map of Mauritania. Mauritania, officially the Islamic Republic of Mauritania, is a sovereign country in northwest Africa. It is bordered by the Atlantic Ocean to the West, western Sahara to the north and the northwest, Algeria to the northeast, Mali to the east and the southeast, and Senegal to the southwest. Mauritania is the 11th largest country in Africa, and 90% of its territory is situated in the Sahara Desert. Most of its population of 4.73 million inhabitants live in the temperate south of the country, with roughly one-third concentrated in the capital and largest city, Nouakchott, located on the Atlantic coast. All study participants were included in the Centre Hospitalier National in Nouakchott.

The epidemiology of HPV infection remains poorly documented in Mauritania [14,24,25,26,27], a country located in northwest Africa with around 5 million inhabitants, one-third being women aged more than 15 years (Figure 1). According to the WHO, with an incidence rate of 14.3% (468 new cases) and a mortality rate of 13.5% (302 deaths) in 2022, cervical cancer ranks as both the 2nd most frequent and most deadly female cancer in Mauritania [14,24,25,26,27]. The estimated age-adjusted death rate associated with cervical cancer is 27.4 per 100,000 Mauritanian women, placing Mauritania at the 20th rank in terms of cervical cancer mortality in the world [28]. Despite this high burden of cervical cancer, molecular epidemiology of circulating HPV genotypes in Mauritania has not yet been reported. Nevertheless, in western Africa, the region Mauritania belongs to, the HPV-related disease burden is high. For instance, in Senegal, a neighboring country, 23.2% of adult women in the general population are estimated to harbor cervical HR-HPV infection [29], and 57.2% [30] to 71.3% [31] of invasive cervical cancers (ICC) are attributed to HPV-16 or HPV-18. Cervical cancer screening and vaccine coverage in Mauritania is currently limited. Prophylactic HPV vaccine is yet available, but the national prevention program has only begun in 2021 [14,24].

We herein conducted a prospective, descriptive study on HPV genotypes distribution in women suffering from high-grade cervical intraepithelial neoplasia (CIN2/3) or ICC. The aim of this study was to assess the HPV epidemiology and the predictive effectiveness of prophylactic HPV vaccination in Mauritania.

## 2. Materials and Methods

### 2.1. Study Design and Participants

A mono-center, descriptive, prospective, population-based study was conducted between 2022 and 2023, in the Centre Hospitalier National, Nouakchott, Mauritania. Over a 12-month period, all adult (≥18-year-old) women followed for suspected high-grade lesions or cervical cancers were recruited after informed consent. Patients with a history of total uterine or cervical resection and those with a history of chemotherapy were excluded. Socio-demographic characteristics including age, educational level, gravidity, parity, menopause, clinical data, and previous HPV screening and vaccination were collected from each patient.

### 2.2. Collection of Biopsy Samples and Processing

Cervical biopsies were performed before treatment for CIN2/3 and ICC through colposcopic examination for histological analysis at the pathology laboratory of the Centre Hospitalier National. The biopsies were fixed in formalin 10% overnight and included in paraffin. The formalin-fixed, paraffin-embedded (FFPE) blocks were further processed using standard histopathological methods and evaluated by a certified pathologist. Histological sections were stained with hematoxylin and eosin (H&E) to identify areas containing abnormal cells. Women diagnosed histologically with either CIN2/3, adenocarcinoma (ADC), or squamous cell carcinoma (SCC) were included in this study. The histopathological examination assessed the representativeness of the biological specimen by evaluating the tumor cellularity with the percentage of tumor cells. The virological analysis was performed accordingly by choosing the most representative inclusion blocks. Depending on the size of the biopsy, five to seven sections of 5–20 µm thick were cut from each FFPE block, placed on a slide for molecular investigations, and sent to the virology laboratory of the Georges Pompidou European Hospital, Paris, France. The patients were staged according to the International Federation of Gynecology and Obstetrics (FIGO) staging system 2009 [32].

### 2.3. DNA Extraction

Sections of FFPE biopsy samples were deparaffinized overnight at +56 °C with 40 µL of proteinase K (Qiagen, Hilden, Germany) and 360 µL of ATL buffer (Qiagen). Afterwards, 200 µL of ATL buffer was added and incubated for 10 min at +70 °C. DNA was further extracted using QIAamp^®^ DNA Mini Kit (Qiagen) according to manufacturer’s instructions and eluted in 100 µL of the kit elution buffer before genotyping. Extracted DNA was stored at −20 °C until analysis.

DNA was quantified using a Qubit^®^ dsDNA BR Assay kit with the Qubit 2.0 fluorimeter (Thermo Fisher Scientific Inc., Waltham, MA, USA).

### 2.4. HPV Detection and Genotyping by Multiplex PCR

HPV detection and genotyping was carried out using the fluorescence-based Bioperfectus Multiplex Real-Time (BMRT) Human Papillomavirus Genotyping Real-Time PCR Kit (Jiangsu Bioperfectus Technologies Co., Ltd., Taizhou, Jiangsu Province, China). The BMRT HPV kit contains primers and corresponding TaqMan probes that amplify a 100-base pairs L1 amplicon, as previously described [33]. According to the HPV classification nomenclature provided by the International Agency for Research on Cancer (IARC) [2], the BMRT HPV Genotyping Real-Time PCR Kit allows to distinguish each of the 21 most prevalent HPV genotypes, including 13 HR-HPV (HPV-16, -18, -31, -33, -35, -39, -45, -51, -52, -56, -58, -59, and -68), 5 possibly oncogenic HPV (HPV-26, -53, -66, -73, and -82), and 3 LR-HPV (HPV-6, -11, and -81). The BMRT HPV kit was used according to the manufacturer’s instructions. Briefly, eight reactions per sample were performed simultaneously. Among them, the reactions A, B, C, D, E, F, and G were prepared to detect and differentiate in FAM™/ VIC^®^ (HEX)/ROX™ fluorescent channels, HPV-16/-18/-31, HPV-59/-66/-53, HPV-33/-58/-45, HPV-56/-52/-35, HPV-68/-51/-39, HPV-73/-26/-82, and HPV-6/-11/-81, respectively. In addition, an internal control (IC) with the housekeeping single-copy gene encoding human DNA topoisomerase III (TOP3) [34], in reaction tube H (FAM™ channel), was set to identify possible PCR inhibition and to confirm the reliability of the reagents in this kit.

PCR amplification was conducted using 96-well reaction plate, in a total reaction volume of 20 μL per well, which comprised 2 μL DNA samples (up to 50 ng), 10 μL of PCR mix containing dUTP instead of dTTP and Uracil-DNA-Glycosylase (UDG), and 8 μL of reaction mix A, B, C, D, E, or F, containing 10 pmol of each primer and 1–5 pmol of each probe (FAM™, VIC^®^, and ROX™ dye). Positive and blank controls were also included in each specimen PCR detection. The reaction plates were sealed with a suitable plastic sheet, centrifuged for 30 s at approximately 2000 revolutions per minute, and subjected to a final PCR amplification. To prevent reamplification of carry-over PCR products, all reactions with UDG were pre-incubated at 50 °C for 5 min, followed by an initial denaturation at 95 °C for 10 min, which also inactivates UDG but activates the DNA polymerase, and 45 cycles at 95 °C for 10 s, 58 °C for 40 s. PCR was performed on the CFX96 real-time PCR instrument (Bio-Rad, Marnes-la-Coquette, France).

The emitted fluorescence was measured by the optical unit of the real-time PCR system. To validate the PCR reaction, the cycle threshold (Ct) values of positive controls had to be less than or equal to the cut-off value of 30.0 in FAM™, VIC^®^ (HEX), and ROX™, and the blank controls had to be undetectable. For each of the 21 detected HPV genotypes, the qualitative reference values of positive cut-off were assessed by the manufacturer using ROC curves based on clinical trial results. Specimens with Ct values less than or equal to the cut-off value of one given HPV type were considered as positive for this HPV genotype. Conversely, specimens with Ct values above the cut-off value of one given HPV type were considered negative. Specimens with Ct values of the internal TOP3 channel above the cut-off value of 30.0 were considered invalid.

The virology laboratory was accredited in 2013 by the Comité Français d’Accréditation (COFRAC) according to the ISO 15189 Norma for the biological markers of specialized molecular medical virology (markers “VIROH”).

### 2.5. Statistical Analysis

Statistical analysis was performed using GraphPad Prism version 8.4.2 (GraphPad Software, Inc., San Diego, CA, USA). Means and standard deviations (SD) were calculated for quantitative variables and proportions for categorical variables. The results were presented along with their 95% confidence interval (CI). Pearson’s χ^2^ or Fisher’s exact tests were used for categorical variables and the non-parametric Mann–Whitney U-test or Kruskal–Wallis’ rank sum test for quantitative variables. Logistic regression models using univariate and multivariate analyses were performed to determine the association of independent variables with HPV type-specific cervical infections (i.e., genital infection with any HPV type and HR-HPV), high-grade lesions and cervical cancer. All statistically significant variables (*p* < 0.05) in the univariate analysis were calculated in a multivariate logistic regression analysis. The crude odds ratio (cOR) and adjusted odds ratio (aOR) were calculated, where appropriate, along with their 95% CI. Finally, a risk factor was defined as an independent variable yielding in the univariate analysis a cOR strictly greater than “1” with a *p*-value less than 0.05. An aOR strictly greater than “1” with a *p* value less than 0.05 defined a risk factor in multivariate analysis.

## 3. Results

### 3.1. Study Population

Out of 85 women enrolled, 68 biopsies were performed in women with CIN or ICC. Biopsies for which socio-demographic information were not precisely known or with insufficient FFPE biopsy material were excluded. Finally, a total of 50 biopsies samples were used in this study. Table 1 presents the socio-demographic characteristics of the study women, the histological diagnosis of cervical lesions, and the distribution of HPV genotypes in cervical biopsy samples.

All women lived in the urban setting of Nouakchott. The mean age of the study population was 56.7 years (range, 35 to 73 years). Most women (40.0%) were aged 60–69 years and the second most frequent age group (26.0%) was women aged 50–59 years. Most participants were engaged in a life couple with a male partner (80.0%), with a low education level (78%), and half of them (48%) had never been to school. All women were multiparous, with a mean number of four children (range, 2 to 6). The majority of them were postmenopausal (84.0%). Only a minority (18.0%) of study women had previously received HPV cervical screening, mainly those diagnosed with CIN2/3 (26.4%). Note that none of the study’s women had ever received prophylactic HPV vaccination and none of them were infected with HIV.

The histological analysis identified 14 (28.0%) CIN2/3 and 36 (72.0%) ICC, including 4 (8.0%) ADC and 32 (64.0%) SCC. Clinical stages I and II were mostly represented with 28.0% and 24.0% of cases, respectively, while extensive cancer (stages III and IV) could be observed in only 1 case out of 5.

### 3.2. Overall Prevalence of HPV and HPV Genotypes Distribution

Molecular detection of the TOP3 gene used as an internal control was conclusive for all samples. A total of 85 interpretable HPV-positive results were detected by the BMRT HPV Genotyping Real-Time PCR Kit in 47 biopsies (94.0%), including 42.5% (*n* = 20) of single HPV genotype infection and 57.5% (*n* = 27) of multiple HPV genotypes infection. Multiple HPV infections were observed in 58.3% of CIN2/3 (7/12), 75.0% of ADC (3/4), and 61.2% of SCC (19/31), without statistical differences in this limited series (*p* = 0.83). The mean number (range) of HPV genotypes detected per sample was 1.58 (1–2) for CIN2/3, 1.75 (1–3) for ADC, and 2.06 (1–4) for SCC, without significant difference between the three categories (*p* = 0.37). A total of 90.6% (77/85) and 3.5% (3/85) of HPV-positive results corresponded to HR-HPV and possibly oncogenic HPV genotypes, respectively, whereas 5.9% (5/85) corresponded to LR-HPV, exclusively HPV-81.

Regarding HPV molecular typing, eleven different genotypes were identified in the 47 biopsy samples (Table 1). The most prevalent HR-HPV genotypes were HPV-45 (*n* = 19; 40.4%), HPV-16 (*n* = 18; 38.3%), HPV-39 and HPV-52 (*n* = 11; 23.4%), HPV-33 (*n* = 8; 17.0%), HPV-18 (*n* = 7; 14.9%), HPV-35 (*n* = 2; 4.2%), and HPV-56 (*n* = 1; 2.1%). Two possibly carcinogenic HPV genotypes were identified, HPV-73 (*n* = 2; 4.2%) and HPV-53 (*n* = 1; 2.1%). Finally, the only LR-HPV was HPV-81 (*n* = 5; 10.6%).

**Figure 2:**
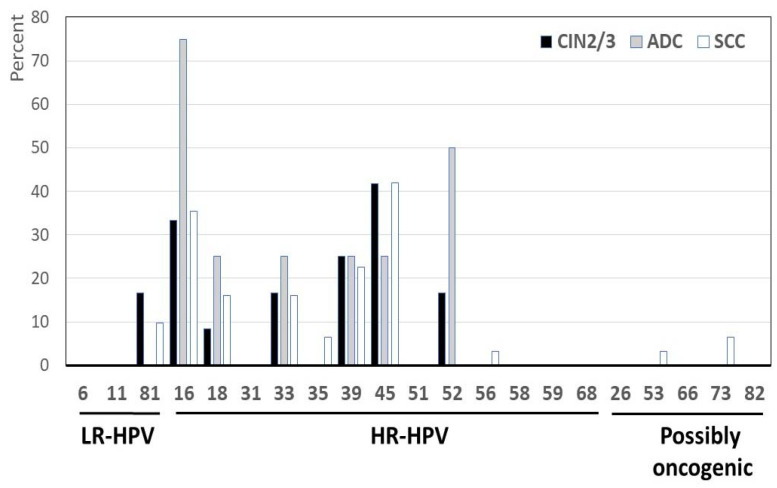
Distribution of HPV types by histology. The samples were classified as high-grade cervical intraepithelial neoplasia (CIN2/3) or invasive cervical cancer, including adenocarcinoma (ADC) or squamous cell carcinoma (SCC). Nota bene: According to the manufacturer’s instructions and in accordance with the HPV classification nomenclature provided by the International Agency for Research on Cancer, the BMRT HPV Genotyping Real-Time PCR Kit distinguishes 21 HPV genotypes, including 14 genotypes (HPV-16, -18, -31, -33, -35, -39, -45, -51, -52, -56, -58, -59, -66, and -68), 3 LR-HPV types (HPV-6, -11, and -81), and 4 genotypes classified as possibly oncogenic (HPV-26, -53, -73, and -82).

Analysis of the different genotypes according to histology is depicted in Figure 2. The most frequent HR-HPV in CIN2/3 were HPV-45 (*n* = 5; 41.7%), HPV-16 (*n* = 4; 33.3%), HPV-39 (*n* = 3; 25.0%), HPV-33,HPV-52 and HPV-81 (*n* = 2; 16.6%) and HPV-18 (*n* = 1; 8.3%); in ADC, HPV-16 (*n* = 3; 75.0%) followed by HPV-18, HPV-33, HPV-39, HPV-45, and HPV-52 (*n* = 1; 25.5%); and in SCC, HPV-45 (*n* = 13; 41.9%), HPV-16 (*n* = 11; 35.5%), HPV-39 (*n* = 7; 22.6%), HPV-18 and HPV-33 (*n* = 5; 16.1%), HPV-81 (*n* = 3;9.7%), HPV-35 and HPV-73 (*n* = 2; 6.4%), and HPV-53 and HPV-56 (*n* = 1; 3.2%). The proportions of the detected HR-HPV genotypes were not different between the respective histological types.

### 3.3. Possible Efficiencies of Cervical HPV Prevention by Gardasil^®^ Vaccines

The potential efficacy of cervical HPV prevention with Gardasil-4^®^ and Gardasil-9^®^ vaccines was further assessed according to the cervical HPV genotypes detected.

Overall, 48.0% (24/50) and 88.0% (44/50) of cervical biopsy samples showed at least one 4-valent and 9-valent HPV vaccine genotype, respectively (Table 1).

Less than 30% (24/85; 28.2%) of the HPV-positive results corresponded to one of the four genotypes covered by the Gardasil-4^®^ vaccine, and none (0.0%) of the cervical biopsy samples contained simultaneously several of these 4-valent vaccine genotypes.

**Figure 3:**
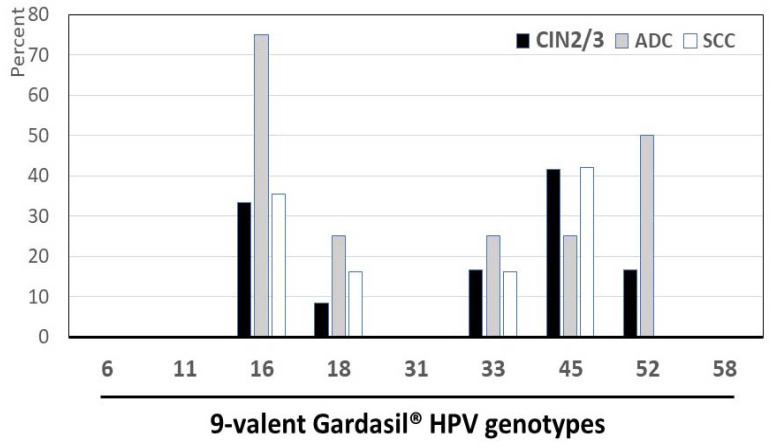
Distribution of HPV genotypes according to their inclusion in the 9-valent Gardasil-9^®^ vaccine: Percentage of low-risk (LR), high-risk (HR), and possibly carcinogenic HPV genotypes in 47 cervical samples positive for HPV DNA by molecular biology according to their possible prevention by Gardasil-9^®^ vaccine among adult women living in Mauritania. Nota bene: The 9-valent Gardasil-9^®^ vaccine (Merck & Co. Inc., Rahway, NJ, USA) targets the seven HR-HPV genotypes predominantly isolated in cervical cancer (HPV-16, -18, -31, -33, -45, -52, and -58) and two LR-HPV (HPV-6 and HPV-11).

The distribution of cervical HPV genotypes according to their inclusion in the 9-valent vaccine is depicted in the Figure 3. For the Gardasil-9^®^ vaccine, 74.1% (63/85) of HPV-positive results corresponded to one HPV type prevented by the 9-valent vaccine (HPV-6, -11, -16, -18, -31, -33, -45, -52, and -58). In addition, 93.6% (44/47) of HPV-positive cervical biopsy samples harbored at least one HR-HPV genotype covered by the Gardasil-9^®^ vaccine, with the majority (26/44; 59.1%) being infected by only one of the 9-valent vaccine HPV genotypes. Moreover, 40.9% (18/44) of these biopsies harbored multiple 9-valent vaccine HPV genotypes simultaneously, including the couples HPV-16 and HPV-45 (*n* = 4), HPV-33 and HPV-45 (*n* = 3), HPV-45 and HPV-52 (*n* = 3), HPV-16 and HPV-33 (*n* = 2), HPV-18 and HPV-45 (*n* = 2), HPV-16 and HPV-52 (*n* = 1), HPV-18 and HPV-52 (*n* = 1), HPV-33 and HPV-58 (*n* = 1), and one biopsy infected with three-vaccine HPV (HPV-16, HPV-45, and HPV-52). Finally, HPV-45 (19/47; 40.4%) and HPV-16 (18/47; 38.9%) were the predominant Gardasil-9^®^ vaccine genotypes, followed by HPV-52 (11/47; 23.4%), HPV-33 (8/47; 17.0%), and HPV-18 (7/47; 14.9%). The Gardasil-9^®^ vaccine HR-HPV types 31 and 58 as well as the LR-HPV types 6 and 11 were not observed.

**Figure 4:**
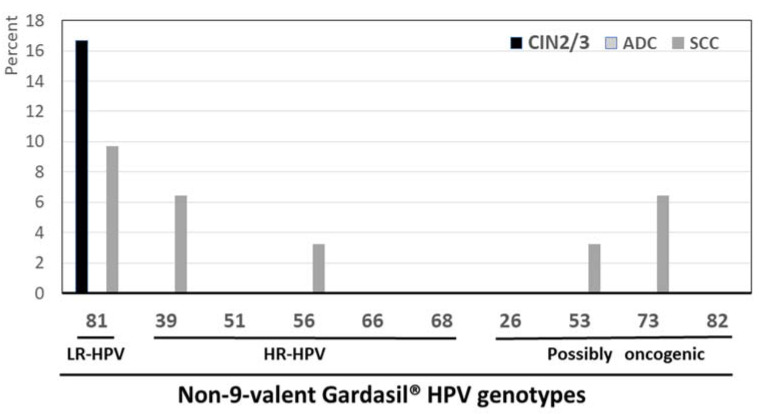
Percentage of low-risk (LR), high-risk (HR), and possibly carcinogenic HPV genotypes in 47 cervical biopsies samples from adult women living in Mauritania, positive for HPV DNA not targeted by the 9-valent Gardasil-9^®^ vaccine by using the BMRT HPV Genotyping Real-Time PCR Kit. Nota bene: The 9-valent Gardasil-9^®^ vaccine (Merck & Co. Inc., Rahway, NJ, USA) targets the seven HR-HPV genotypes predominantly isolated in cervical cancer (HPV-16, -18, -31, -33, -45, -52, and -58) and two LR-HPV (HPV-6 and HPV-11).

A total of 25.9% (22/85) of HPV-positive results corresponded to HPV genotypes not covered by the Gardasil-9^®^ vaccine, including HPV-39 (11/47; 23.4%), HPV-81 (5/47; 10.6%), HPV-35 (2/47; 4.2%), HPV-73 (2/47; 4.2%), HPV-53 (1/47; 2.1%), and HPV-56 (1/47; 2.1%). Therefore, the majority of nonvaccine types were HR-HPV (14/22; 63.6%) (Figure 4). Thus, among all detected HR-HPV types, HPV-39, HPV-35, and HPV-56, representing 16.5% (14/85) of HPV-positive results, were not targeted by the Gardasil-9^®^ vaccine. In other terms, the majority (5/8; 62.5%) of the eight detected HR-HPV genotypes were covered by the 9-valent HPV vaccine, while the remaining (37.5%) corresponded to nonvaccine types. Finally, nonvaccine HR-HPV detected in biopsy samples were frequently co-infected with vaccine-targeted HR-HPV in 64.3% (9/14).

### 3.4. Determinants of HPV Outcomes and High-Grade Cervical Lesions and Invasive Carcinoma Using Logistic Regression Analyses

To identify factors that determine the HPV infection profile and the occurrence of high-grade cervical lesions and invasive cervical carcinoma among these Mauritanian women, sociodemographic and clinical characteristics of the participants and the distribution of HPV genotypes were calculated in simple and multivariate logistic regression analyses. The results are presented in Table 2, Table 3 and Table 4.

#### 3.4.1. Simple Logistic Regression Analysis

Older women, particularly those aged 60–69 years old, had increased likelihood of being infected with several HR-HPV genotypes (cOR: 3.21, 95% CI: 1.01–10.95, *p* = 0.048), especially those high-risk genotypes targeted by the Gardasil-9^®^ vaccine (cOR: 4.02, 95% CI: 1.22–14.30, *p* = 0.022) (Table 2). On the other hand, these women aged 60–69 years old showed a reduced risk of having CIN2/3 cervical lesions (cOR: 0.16, 95% CI: 0.03–0.72, *p* = 0.015) (Table 4). In addition, having stopped schooling at high-school level was associated with an increased risk of being both infected with possibly oncogenic HPV genotypes (cOR: 7.8, 95% CI: 1.18–54.18, *p* = 0.034) as well as harboring CIN2/3 lesions (cOR: 6.11, 95% CI: 1.26–34.78, *p* = 0.024) (Table 2 and Table 4). Regarding the childbearing status of the women, while having had at least six pregnancies was associated with a reduced risk of being infected with nonvaccine HR-HPV genotypes (cOR: 0.154, 95% CI: 0.01–0.92, *p* = 0.038), having had up to three pregnancies was associated with an increased likelihood of harboring CIN2/3 lesions (cOR: 6.8, 95% CI: 1.15–54.58, *p* = 0.034), (Table 2 and Table 4). In addition, having given birth up to four times was significantly associated with an increased risk of being infected with several HR-HPV genotypes (cOR: 4.09, 95% CI: 1.26–14.55, *p* = 0.017) (Table 2). Concerning the impact of the stages of the cervical lesions and cancer, the FIGO stages IA/B were associated with a reduced risk of being infected with either several HPV genotypes (cOR: 0.136, 95% CI: 0.03–0.53, *p* = 0.003), several HR-HPV genotypes (cOR: 0.195, 95% CI: 0.04–0.75, *p* = 0.016), or several high-risk genotypes targeted by the Gardasil-9^®^ vaccine (cOR: 0.208, 95% CI: 0.03–0.91, *p* = 0.036) (Table 2). Interestingly, while women exhibiting FIGO stages IA/B had an increased likelihood of harboring a cervical ADC (cOR: 9.54, 95% CI: 1.09–204.2, *p* = 0.041), those exhibiting neoplasia with FIGO stages IIA/B were more likely to have cervical squamous cell carcinoma (cOR: 8.91, 95% CI: 1.5–171.0, *p* = 0.013) (Table 4). On the other hand, women having neoplasia with FIGO stages IIIA/B were more likely to carry HR-HPV genotypes targeted by the Gardasil^®^ vaccine (cOR: 9.33, 95% CI: 1.47–182.6, *p* = 0.015) (Table 2). Women who reported having had previous HPV screening had higher likelihood of harboring CIN2/3 lesions (cOR: 8.25, 95% CI: 1.79–46.46, *p* = 0.006), while showing a reduced risk of harboring SCC (cOR: 0.21, 95% CI: 0.04–0.92, *p* = 0.037) (Table 4). The younger age, marital status, and the clinical symptoms did not show any association with any of the HPV outcomes and cervical high-grade lesions and cancers in the simple logistic regression analyses. Likewise, the HPV genotypes distribution did not show any association with either CIN2/3 lesions, SCC or ADC.

#### 3.4.2. Multivariate Logistic Regression Analysis

Only the independent variables with a statistically significant association (*p* < 0.05) in the simple logistic regression analysis were computed in the multivariate logistic regression (Table 3 and Table 4). Women aged 60–69 years old had an increased risk of being infected with HR-HPV genotypes targeted by the Gardasil-9^®^ vaccine (aOR: 4.25, 95% CI: 1.17–15.42, *p* = 0.028). Likewise, those who had stopped schooling at high-school level were more at risk of being infected with possibly oncogenic HPV genotypes (aOR: 7.8, 95% CI: 1.22–49.67, *p* = 0.03) (Table 3). In addition, women who reported having given birth up to four times had a significant increased risk of being infected with several HR-HPV genotypes (aOR: 4.22, 95% CI: 1.10–16.10, *p* = 0.035) (Table 3). Regarding the FIGO stages of cervical cancer, stages IA/B remained associated with a reduced likelihood of being infected with several HPV genotypes (aOR: 0.136, 95% CI: 0.03–0.58, *p* = 0.007) and HR-HPV genotypes (aOR: 0.16, 95% CI: 0.03–0.77, *p* = 0.022) (Table 3). On the other hand, women with cervical neoplasia of stages IIA/B and IIIA/B had significantly high likelihoods of harboring SCC (aOR: 11.12, 95% CI: 1.12–109.99, *p* = 0.039) and Gardasil^®^ vaccine genotypes (aOR: 9.33, 95% CI: 1.05–82.78, *p* = 0.045), respectively (Table 3 and Table 4). Interestingly, while the high likelihood of harboring CIN2/3 lesions which was associated with reporting having previously been screened for HPV was lost in the multivariate regression analysis, the protective effect of a history of previous HPV screening against the occurrence of SCC was significantly confirmed in the multivariate analysis (aOR: 0.16, 95% CI: 0.03–0.94, *p* = 0.043) (Table 4).

## 4. Discussion

We herein evaluated for the first time the HPV genotypes distribution in Mauritanian women suffering from CIN2/3 or ICC, with the perspective of prophylactic vaccination against HPV. The results showed that single or multiple HR-HPV genotypes were generally detected by molecular biology in cervical biopsy material from CIN2/3, ADC, and SCC tissues. Interestingly, only a limited number of HR-HPV types, including mainly HPV-45, HPV-16, HPV-39, HPV-52, and HPV-33, were detected. This distribution possibly reflects a specific molecular epidemiology signature of HR-HPV associated with cervical lesions in Mauritania. In addition, the majority (88% of all biopsies and 93.6% of HPV-positive results) of study women suffering from severe cervical dysplasia, ADC, or SCC were infected by vaccine HR-HPV genotypes, including mostly HPV-45, HPV-16, HPV-52, HPV-33, and HPV-18. However, other aggressive nonvaccine HR-HPV genotypes were also detected, indicating their circulation in the adult female population in Mauritania. Taken together, our observations confirm the association of HR-HPV with the genesis of precancerous and cancerous cervical lesions in Mauritanian women. Undoubtedly, these observations indicate the existence of a specific epidemiological profile with highly pathogenic HR-HPV types circulating in the country. These findings also demonstrate the frequent involvement of nonvaccine HR-HPV genotypes, in cervical precancerous and cancerous lesions, mostly in co-infection with vaccine HR-HPV types. On public health perspective, our findings point out that prophylactic HPV vaccination must be necessarily combined with primary molecular screening of cervical HR-HPV infection. Combining these two preventive strategies would help in reaching the objectives of the WHO global strategy to accelerate the elimination of cervical cancer in 2030 [17], and ultimately to eradicate the second most prevalent female cancer in Mauritania.

Geographical distribution of HPV genotypes provides specific molecular epidemiology of circulating HPV strains that varies according to regions and countries. As a first approach, we evaluated the distribution of HPV genotypes associated with high-grade lesions or ICC in women living in Mauritania. HPV DNA was detected in the majority (94.0%) of cervical biopsies from study women presenting with CIN2/3, ADC, and SCC, with at least one HR-HPV in all HPV-positive samples and frequent (48.0%) multiple HR-HPV infections. Similar high prevalence rates of cervical HPV and HR-HPV infections are generally reported in adult women living in sub-Saharan Africa suffering from HPV-related cervical lesions, with wide variations between regions or countries, ranging from 85.9% in Senegal [31], 90.4% in Ghana, Nigeria, and South Africa [21], 92.4% [35] and 100% [36] in Gabon, 94.1% in Ethiopia and the Sudan [37], 100% in Zambia [38] and in Republic of Congo [39].

The diversity of HR-HPV genotypes detected in FFPE biopsy samples from Mauritanian women appeared limited, as previously reported in other studies conducted in FFPE cervical high-grade tumor biopsies in sub-Saharan Africa. Thus, only six oncogenic genotypes were detected in study women (HPV-45, HPV-16, HPV-39, HPV-52, and HPV-33). In Gabon, the diversity of oncogenic HPV in FFPE cervical biopsies was similarly limited to six genotypes (HPV-16, HPV-18, HPV-33, HPV-35, HPV-45, and HPV-58) [35], whereas nine HR-HPV genotypes could be detected in Senegal (HPV-16, HPV-18, HPV-31, HPV-33, HPV-35, HPV-39, HPV-45, HPV-56, and HPV-58) [31] and Cameroon (HPV-16, HPV-18, HPV-33, HPV-35, HPV-39, HPV-45, HPV-51, HPV-56, and HPV-82) [40]. The preselection of cervical biopsy samples with CIN2/3 or ICC decreases the probability of finding HPV genotypes not associated with cancer or high-grade lesions. Another factor that could reduce the diversity of HPV infection that could be envisaged in these women is the fact that all male partners in Mauritania are ritually circumcised in childhood [41]. Our observations point out also that the diversity of oncogenic HPV varies among sub-Saharan African countries. Indeed, the two main genotypes detected in CIN or ICC worldwide are HPV-16 and HPV-18 [42]. For example, in Gabon, the prevalence of HPV-16 was 75.4% and that of HPV-18 was 18.0% [35]. In Senegal, the prevalence of HPV-16 was 56.8% and that of HPV-18 was 14.6% [31]. Thus, the distribution of oncogenic HR-HPV genotypes in Mauritania appears unique, with HPV-16 and HPV-18 which ranked only as the second and sixth most detected genotypes, respectively. The most prevalent genotype was HPV-45, and the oncogenic genotypes HPV-39, HPV-52, and HPV-33 were more prevalent than HPV-18. The prevalence of HPV-45 (40.4%) was the highest in our series, which is reminiscent to the relatively high prevalence (13.0%) of this genotype in cervical lesions in Senegal and more generally in Africa (7.2% to 15.5%) [21,22,23]. Like what was observed for HPV-45, the genotype HPV-16, which was the second (38.3%) most detected in Mauritania women, is also frequently detected in ICC in sub-Saharan Africa, ranging from 13.8% [23], 51.2% [21] to 58.2% [22]. In addition, HPV-52, the third (23.4%) most detected genotype in our study, is also commonly detected in HPV-associated cervical lesions in the eastern and southern African areas [23], with large variations ranging from 2.3% to 13.7% [22]. However, in Senegal, while genital carriage of HPV-52 was very common in the general population (3.5%) [29] and female sex workers (32.6%) [43], this genotype was rarely detected in ICC in Mali and Senegal (0.7%) [30]. In Caucasian women, some HPV-52 variants have been described as being associated with a higher risk of developing high-grade lesions or cervical cancer [44,45]. Further genomic polymorphism studies of HPV-52 strains might be helpful to better understand the mechanisms of persistence or elimination of HPV-52 and its association with CIN or ICC in Mauritanian women [45]. Among LR-HPV, HPV-81 was detected in ten percent of study women, whereas the HPV-6 and HPV-11 targeted by the Gardasil^®^ HPV vaccines could not be found. HPV-81 was relatively frequently detected in one of ten women with CIN2/3 or SCC, a prevalence of a similar range to those previously reported in childbearing-aged women living in Kenya (7.4%) [46] and South Africa (7.6%) [47]. According to IARC, HPV-81 is classified as an LR-HPV [2]. HPV-81 is recognized as a new type mostly found in individuals with a weak immune system [48], which is also a risk factor associated with precancerous and cancerous lesions [46,49]. However, none of our study participants were infected with HIV. On the other hand, HPV-81 was always associated with one or two HR-HPV types in our study, and its involvement in carcinogenesis remains uncertain. Whether HPV-81 may play a role in cancer etiology as a syndemic partner likely warrants further investigations.

A high rate (54.0%) of multiple HPV infection (2 to 3 HPV types) was observed in our study population. This proportion is higher than that generally found in other series which varied from 4% to 39% depending on the clinical stages [21,31,50,51,52,53,54] and HIV-serostatus [21,22,55]. In Senegal, a very high rate of multiple HPV genital infections (70.1%) was reported in female sex workers [43]. Whether multiple genital HPV infections in Mauritanian women could be associated with high-risk sexual behavior remains unknown. Otherwise, the association between multiple HPV infections and HIV infection previously reported [56,57] cannot explain the high prevalence of multiple cervical HPV infections in our study women, as they were not infected with HIV. Regardless, these features may constitute a real concern for the health care of Mauritanian women as multiple cervical HR-HPV infections increase the risk of cervical abnormalities [58], and are associated with poor outcome and treatment response of invasive cancer [52,59].

Cervical cancer is the leading cause of cancer-related deaths among women in sub-Saharan Africa, but it can be prevented by vaccination [9]. Although current vaccines only target up to nine different genotypes, cross-immunity and probably cross-protection occur between the genotypes targeted by the vaccine and nonvaccine genotypes [60]. However, the effectiveness of cervical HPV prevention by prophylactic vaccines remains largely dependent on the molecular epidemiology of HPV strains present in each region, most particularly in women with high-grade lesions or ICC. The majority (88.0%) of study women suffering from CIN2/3 or ICC were infected by 9-valent HPV Gardasil-9^®^ vaccine genotypes. Furthermore, most (93.6%) women with HPV-positive cervical samples harbored at least one 9-valent HPV genotype. HPV-45 was the predominant genotype, followed by HPV-16, HPV-52, HPV-33, and HPV-18, and 38.3% of women were infected by multiple (two to three) 9-valent vaccine HPV genotypes. When considering all HPV-positive results, the majority (74.1%) corresponded to HPV genotypes covered by the Gardasil-9^®^ vaccine, while 25.9% corresponded to nonvaccine HPV genotypes, mainly nonvaccine HR-HPV (HPV-39, HPV-35, and HPV-56). Detection of nonvaccine HR-HPV types circulating in the general adult female population, which may be non-rarely detected in precancerous and cancerous cervical tissues, is frequently reported in several sub-Saharan African countries [22,31,61,62,63]. Two-thirds of biopsies with nonvaccine HR-HPV were co-infected with vaccine-targeted HR-HPV, as previously observed in Ghana for HPV-35 and HPV-56 [61]. The co-infection of vaccine genotypes with nonvaccine HR-HPV may reduce the efficacy of HPV vaccines in preventing cervical cancer [22]. It has been suggested that the high prevalence of cervical HPV infections harboring simultaneously both nonvaccine HR-HPV and vaccine HR-HPV could be associated with the high age-standardized incidence rate (ASIR) of cervical cancer in sub-Saharan Africa [22]. Taken together, our observations in Mauritanian women indicate that around 90% of CIN2/3, ADC, or SCC are associated with HR-HPV covered by the 9-valent Gardasil^®^ HPV vaccine. However, these findings also highlight the presence of other aggressive nonvaccine HR-HPV types representing one-third of oncogenic HR-HPV detected, indicating indirectly their circulation in the adult female population living in Mauritania. Our observations raise several practical issues in the prevention of HPV infection and cervical cancer in Mauritania. Firstly, the prophylactic HPV vaccination, which likely covers the majority of genital HPV detected in adult Mauritanian women suffering from severe cervical dysplasia, ADC, or SCC, remains essential for the primary prevention of cervical cancer. Thus, a public health program involved in HPV vaccination implementation should be clearly extended at the national level. However, according to our observations, prophylactic HPV vaccination would remain insufficient to cover all HPV genotypes associated with precancerous or malignant cervical lesions that might represent a risk for cervical cancer in Mauritania. Secondly, it now seems necessary to design for Mauritania and neighboring countries, as for many other countries in sub-Saharan Africa, a prophylactic HPV vaccine better adapted to the viral strains of HPV circulating in sub-Saharan regions in women after age 30 (or after age 25 if living with HIV). Finally, continuation and generalization of primary molecular screening of carcinogenic HPV according to the recently revised WHO recommendations [17] as well as the implementation of pathology laboratory facilities for cytology and histological confirmation and triage of HR-HPV-positive cases, which remains challenging in sub-Saharan Africa [64], are necessary to eradicate cervical cancer in Mauritania. New prevention strategies for cervical carcinoma, particularly those using self-sampling of female genital secretions for HPV molecular testing, are particularly suited to resource-constrained settings for hard-to-reach populations or when medical facilities are lacking [65,66,67,68,69].

In the present series, the number of SCC was about 10 times higher than ADC, as previously reported in the literature [39,70,71]. According to sociological aspects, our population of patients with CIN2/3 lesions or cervical cancer consisted of women ranging from 35 to 73 years old (mean: 56.7 years). Almost two-thirds of HPV-positive patients were over 50 years old, and the most affected age group was 60–69 years old. Stages II to IV were frequently represented (44.0%), testifying to a delay in screening, which may be linked to a lack of sensitization in women whose level of education was extremely low with half of the women never having been to school. Very similar socio-demographic profiles and stages of HPV-related lesions were previously observed in the neighboring country Senegal [31]. The multivariate analysis of the different variables of interest is difficult to interpret, due to the small size of the included population. However, it appears that advanced age and clinical stage were associated with multiple HR-HPV infections, likely because of immune-senescence or tumor aggressiveness [72,73]. Previous HPV screening was associated with precancerous CIN2/3 diagnosis and decreased occurrence of SCC, which confirms the critical need for effective primary molecular HPV screening in adult women living in Mauritania, with particular attention for elderly women.

No HPV DNA sequences were identified in 6.0% of our cases despite retesting. This rate of HPV-negative biopsy samples is lower than those generally reported for the FFPE sample, ranging from 10% [74], 10.8% [75], 12.5% [30] to 14.1% [31]. In our study, the quality of the DNA was assessed by evaluating the housekeeping TOP3 gene in all samples, which were all found to be adequate. However, negative HPV results could have been related to the sampling method. Indeed, FFPE preparation for molecular analysis or DNA extraction is composed of several critical steps that could impact the amount or the quality of genetic material to be amplified [76]. Indeed, it is well reported that DNA recovering in FFPE specimens may be partially degraded, influenced by several factors, such as formalin quality and concentration, length of fixation, paraffin quality, and temperature [76]. Consequently, DNA in FFPE biopsies is either completely or partially degraded into DNA fragments of 200 bp or less [77,78]. Interestingly, the Bioperfectus HPV assay relies on the amplification of only 100 bp-fragments, a feature that could be associated with better PCR efficiency when using DNA extracted from FFPE biopsies, and which could partly explain the low rate of HPV-negative samples in this study. It is also possible that mutations affecting the priming sites of the primers used in the Bioperfectus assay could have led to misdetection. Furthermore, the target region of the multiplex PCR used may have been deleted during the process of integration of the viral DNA into the host genome that occurs in more than 80% of cervical cancers [79,80]. Finally, the possibility that a small proportion of cervical carcinoma develops independently of HPV oncogenesis must be considered regarding the policy of vaccination and of detection of intraepithelial neoplasia based on virological testing [31].

Our study has several limitations that need to be considered. First, the representativeness of the included study population is not ensured. Indeed, such inclusion bias could be extended in other urban and rural remote areas in Mauritania, where no women were included. Second, the small sample size of our study population may have introduced a selection bias because included precancerous and cancer cases could represent a select group of women who were healthy enough to participate in study enrollment, and therefore likely had less severe cancer stages. Third, participants were included on a voluntary basis, which may constitute a source of recruitment bias, impacting the validity of the answers to the sociodemographic questionnaire, including items related to the intimacy of their lives. Finally, the preselection of pathologic cervical biopsy material decreases the probability of finding HPV genotypes not associated with high-grade lesions or cancer. Furthermore, FFPE biopsy samples are not the ideal material for HPV detection.

## 5. Conclusions

Our study shows that the HR-HPV genotypes in CIN2/3 and cervical cancer in Mauritanian women match generally with the HPV types targeted by the prophylactic 9-valent Gardasil-9^®^ HPV vaccine, but unfrequently with the Gardasil-4^®^ vaccine. This feature highlights the need for a shift from the quadrivalent to the 9-valent HPV vaccine for protection against chronic HPV infection and cervical neoplasia in Mauritania. The high prevalence of HPV types targeted by the Gardasil-9^®^ HPV vaccine supports the continuation of the 9-valent HPV vaccine national program as this vaccine targets a large number of HR-HPV types that cause cancer. Our results also strongly support the intensification of a largescale roll-out of HPV vaccination to Mauritanian girls and the establishment of catch-up campaigns for young adolescents or adults. The reinforcement of primary molecular HPV testing and pathological triage are essential for the early detection of cervical precancerous lesions among women who are already infected with HPV, unvaccinated or partially vaccinated, and those infected with HPV types not targeted by current HPV vaccines. The circulation of nonvaccine HR-HPV types (16.5%), specifically HPV-39, HPV-35, and HPV-56, also raises concern in the Mauritanian population. In addition, our study showed that a minority of CIN2/3 or ICC might remain HPV-negative. Taken together, our observations could help inform health policy makers by providing useful HPV baseline data for assessing the impact of HPV vaccine effectiveness, and possibly assist in the development of a policy to improve HPV vaccination strategies. Finally, the primary prevention of precancerous and cancerous cervical diseases in Mauritania by the 9-valent prophylactic vaccination should be integrated into a large national program including sensitization and screening to sustainably decrease the mortality rate related to cervical cancer.

## Figures and Tables

**Table 1:** Socio-demographic characteristics, histological diagnosis of cervical lesions, and distribution of HPV types in the study’s 50 adult women suffering from cervical intraepithelial neoplasia or invasive cervical cancer, and living in the urban setting of Nouakchott, the capital city of Mauritania.

	All Women (*n* = 50)	CIN2/3 (*n* = 14)	ADC (*n* = 4)	SCC (*n* = 32)
Characteristics [*n* (%) [95% CI] *]				
Age (Years)				
**All ages** [Mean (SD)]	56.7 (9.4)	51.6 (10.3)	58.5 (9.5)	58.9 (8.3)
30–39	3(6.0) [0.0–12.6]	2(14.3) [0.0–32.6]	0 (0.0) NA	1 (3.1) [0.0–9.1]
40–49	9 (18.0) [7.3–28.6]	4 (28.6) [4.9–52.2]	1 (25.0) [0.0–67.4]	4 (12.5) [1.0–23.9]
50–59	13 (26.0) [13.8–38.1]	5 (35.7) [10.6–60.8]	0 (0.0) NA	8 (25.0) [10.0–40.0]
60–69	20 (40.0) [26.4–53.3]	2 (14.3) [0.0–32.6]	3 (75.0) [32.6–100.0]	15 (46.9) [29.6–64.2]
≥70	5 (10.0) [1.7–18.3]	1 (7.2) [0.0–20.6]	0 (0.0) NA	4 (12.5) [1.0–23.9]
Marital status [*n* (%) CI]				
Living in couple or married	40 (80.0) [68.9–91.1]	13 (92.8) [79.4–100.0]	2 (50.0) [1.0–99.0]	25 (78.1) [63.8–92.4]
Divorced/widow	10 (20.0) [8.9–31.1]	1 (7.2) [0.0–20.6]	2 (50.0) [1.0–99.0]	7 (21.9) [7.5–36.2]
Education level [*n* (%) CI]				
Never schooled	24 (48.0) [34.1–61.8]	5 (35.7) [10.6–60.8]	1 (25.0) [0.0–67.4]	18 (56.2) [39.0–73.4]
Elementary	15 (30.0) [17.3–42.7]	2 (14.3) [0.0–32.6]	3 (75.0) [32.6–100.0]	10 (31.2) [15.2–47.3]
High school	8 (16.0) [5.8–26.2]	5 (35.7) [10.6–60.8]	0 (0.0) NA	3 (9.4) [0.0–19.5]
University	3 (6.0) [0.0–12.6]	2 (14.3) [0.0–32.6]	0 (0.0) NA	1 (3.1) [0.0–9.1]
Gravidity [*n* (%) CI]				
1–3	6 (12.0) [2.9–21.0]	4 (28.6) [4.9–52.2]	0 (0.0) NA	2 (6.2) [0.0–14.6]
4–5	31 (62.0) [48.5–75.4]	7 (50.0) [23.8–76.2]	3 (75.0) [32.6–100.0]	21 (65.6) [49.2–82.1]
≥6	13 (26.0) [13.8–38.1]	3 (21.4) [0.0–42.9]	1 (25.0) [0.0–67.4]	9 (28.2) [12.5–43.7]
Parity [*n* (%) CI]				
1–3	14 (28.0) [15.5–40.4]	5 (35.7) [10.6–60.8]	1 (25.0) [0.0–67.4]	8 (25.0) [10.0–40.0]
4–5	35 (70.0) [57.3–82.7]	8 (57.1) [31.2–83.1]	3 (75.0) [32.6–100.0]	24 (75.0) [60.0–90.0]
≥6	1 (2.0) [0.0–5.9]	1 (7.2) [0.0–20.6]	0 (0.0) NA	0 (0.0) NA
Menopause [*n* (%) CI]				
Yes	42 (84.0) [73.8–94.1]	10 (71.4) [47.8–95.1]	3 (75.0) [32.6–100.0]	29 (90.6) [80.5–100.0]
No	8 (16.0) [5.8–26.1]	4 (28.6) [4.9–52.2]	1 (25.0) [0.0–67.4]	3 (9.4) [0.0–19.5]
FIGO Staging [*n* (%) CI]				
[0]	14 (28.0) [15.5–40.5]	14 (100.0) NA	0 (0.0) NA	0 (0.0) NA
IA/B	14 (28.0) [15.5–40.5]	0 (0.0) NA	3 (75.5) [32.6–100.0]	11 (34.4) [17.9–50.8]
IIA/B	12 (24.0) [12.1–35.8]	0 (0.0) NA	1 (25.0) [0.0–67.4]	11 (34.4) [17.9–50.8]
IIIA/B	8 (16.0) [5.8–26.1]	0 (0.0) NA	0 (0.0) NA	8 (25.0) [10.0–40.0]
IVA/B	2 (4.0) [0.0–9.4]	0 (0.0) NA	0 (0.0) NA	2 (6.2) [0.0–14.6]
HPV DNA detection and genotypes [*n* (%) CI]				
HPV DNA	47 (94.0) [87.4–100.0]	12 (85.7) [67.4–100.0]	4 (100.0) NA	31 (96.9) [90.8–100.0]
Multiple types of any HPV	27 (54.0) [53.4–67.7]	7 (50.0) [23.8–76.2]	2 (50.0) [1.0–99.0]	18 (56.2) [39.0–73.4]
LR-HPV	5 (10.0) [1.7–18.3]	2 (14.3) [0.0–32.6]	0 (0.0) NA	3 (9.4) [0.0–19.5]
HR-HPV	47 (94.0) [87.4–100.0]	12 (85.7) [67.4–100.0]	4 (100.0) NA	31 (96.9) [90.8–100.0]
Multiple types of HR-HPV	24 (48.0) [34.1–61.8]	5 (35.7) [10.6–60.8]	2 (50.0) [1.0–99.0]	17 (53.1) [35.8–70.4]
Possibly oncogenic HPV	3 (6.0) [0.0–12.6]	0 (0.0) NA	0 (0.0) NA	3 (9.4) [0.0–19.5]
HPV-16	18 (36.0) [22.7–49.3]	4 (28.6) [4.9–52.2]	3 (75.0) [32.6–100.0]	11 (34.4) [17.9–50.8]
HPV-18	7 (14.0) [4.4–23.6]	1 (7.1) [0.0–20.6]	1 (25.0) [0.0–67.4]	5 (15.6) [3.0–28.2]
Any 4-valent vaccine types **	24 (48.0) [34.1–61.8]	5 (35.7) [10.6–60.8]	3 (75.0) [32.6–100.0]	16 (50.0) [32.7–67.3]
Multiple 4-valent vaccine types	0 (0.0) NA	0 (0.0) NA	0 (0.0) NA	0 (0.0) NA
Any 9-valent vaccine types ***	44 (88.0) [78.9–97.0]	11 (78.6) [57.1–100.0]	4 (100.0) NA	29 (90.6) [80.5–100.0]
Multiple 9-valent vaccine types	18 (36.0) [22.7–49.3]	4 (28.6) [4.9–52.2]	1 (25.0) [1.0–99.0]	9 (28.1) [20.7–54.3]
Nonvaccine HR-HPV types	14 (28.0) [15.5–40.4]	3 (35.7) [0.0–48.1]	2 (50.0) [0.0–67.4]	10 (31.2) [15.2–48.9]

* The 95% confidence intervals are presented in brackets; ** The 4-valent Gardasil-4^®^ vaccine (Merck & Co. Inc., Rahway, NJ, USA) is effective against HPV genotypes -6, -11, -16, and -18; *** The 9-valent Gardasil-9^®^ vaccine (Merck & Co. Inc.) is effective against HPV genotypes -6, -11, -16, -18, 31, -33, -45, -52, and -58. Nota bene: HPV DNA detection and genotypes were from all 50 women in the study. ADC: Adenocarcinoma; 95% CI: 95% Confidence interval; CIN2/3: Cervical Intraepithelial Neoplasia 2/3; FIGO: International Federation of Gynecology and Obstetrics; High-grade cervical intraepithelial neoplasia; HPV: Human papillomavirus; HR-HPV: High-risk human papillomavirus; LR-HPV: Low-risk HPV:; NA: Not applicable; SCC: squamous cell carcinoma; SD: Standard deviation.

**Table 2:** Risk factors associated with HPV outcomes using simple logistic regression analysis in 50 adult women living in Nouakchott, Mauritania.

	HPV		Multiple HPV		HR-HPV		Multiple HR-HPV		^&^ Gardasil^®^ HR-HPV		^£^ Gardasil-9^®^ HR-HPV		Multiple Gardasil-9^®^ HR-HPV		Nonvaccine HR-HPV		Possibly Oncogenic HPV	
	cOR (95% CI)	*p* ^a^	cOR (95% CI)	*p*	cOR (95% CI)	*p*	cOR (95% CI)	*p*	cOR (95% CI)	*p*	cOR (95% CI)	*p*	cOR (95% CI)	*p*	cOR (95% CI)	*p*	cOR (95% CI)	*p*
**Age**																		
30–39	NC ^b^	NC	1.76 (0.16–39.38)	0.645	NC	NC	2.273 (0.21–50.86)	0.501	2.087 (0.18–46.69)	0.547	0.238 (0.02–5.65)	0.313	0.882 (0.04–9.87)	0.920	5.833 (0.52–132.3)	0.149	4.2 (0.17–52.59)	0.313
40–49	0.411 (0.03–9.44)	0.509	0.354 (0.06–1.54)	0.168	0.411 (0.03–9.44)	0.509	0.476 (0.09–2.06)	0.326	0.432 (0.08–1.87)	0.265	1.111 (0.15–22.81)	0.927	0.446 (0.06–20.13)	0.327	0.691 (0.09–3.37)	0.664	2.643 (0.32–16.61)	0.332
50–59	0.685 (0.06–15.52)	0.771	0.426 (0.11–1.53)	0.191	0.685 (0.06–15.52)	0.770	0.377 (0.08–1.38)	0.143	0.812 (0.22–2.90)	0.747	1.875 (0.26–37.85)	0.562	0.44 (0.08–1.72)	0.247	1.2 (0.27–4.65)	0.797	0.533 (0.03–3.77)	0.563
60–69	NC	NC	20.122 (0.67–7.08)	0.200	NC	NC	3.208 (1.01–10.95)	**0.048**	2.786 (0.88–9.41)	0.081	1.385 (0.24–10.76)	0.719	4.016 (1.22–14.30)	**0.022**	0.777 (0.21–2.74)	0.698	0.722 (0.09–40.12)	0.719
≥70	0.186 (0.02–4.51)	0.249	3.826 (0.52–77.97)	0.201	0.186 (0.02–4.51)	0.249	0.697 (0.08–4.59)	0.704	0.218 (0.01–1.62)	0.144	0.5 (0.57–10.80)	0.587	0.412 (0.02–3.07)	0.412	0.615 (0.03–4.67)	0.665	NC	NC
**Education level**																		
Never	0.44 (0.02–4.89)	0.502	2.727 (0.88–8.95)	0.082	0.44 (0.02–4.89)	0.501	1.612 (0.53–5.03)	0.401	1.00 (0.33–3.05)	>0.99	0.913 (0.15–5.41)	0.916	1.607 (0.51–5.26)	0.422	0.75 (0.21–2.59)	0.649	NC	NC
Elementary	0.848 (0.07–19.12)	0.897	0.444 (0.12–1.51)	0.193	0.848 (0.07–19.12)	0.897	0.629 (0.17–2.12)	0.457	1.21 (0.36–4.16)	0.757	0.838 (0.14–6.58)	0.850	0.846 (0.22–2.95)	0.796	0.545 (0.11–2.15)	0.399	2.667 (0.44–16.24)	0.272
High school	NC	NC	0.826 (0.17–3.92)	0.804	NC	NC	1.1 (0.23–5.22)	0.901	0.545 (0.10–2.51)	0.438	0.945 (0.12–19.56)	0.962	1.08 (0.19–5.04)	0.923	3.2 (0.65–15.96)	0.147	7.8 (1.18–54.18)	**0.034**
University	NC	NC	0.403 (0.02–4.49)	0.457	NC	NC	0.522 (0.02–5.81)	0.596	2.087 (0.18–46.69)	0.547	NC	NC	NC	NC	1.308 (0.05–14.81)	0.834	NC	NC
**Marital status**																		
Living in couple	NC	NC	0.226 (0.03–1.04)	0.056	NC	NC	0.316 (0.06–1.32)	0.116	0.351 (0.06–1.46)	0.152	NC	NC	0.807 (0.19–3.61)	0.769	0.885 (0.20–4.66)	0.875	1.286 (0.17–26.24)	0.824
Divorced/ Widowed	NC	NC	4.421 (0.96–31.78)	0.056	NC	NC	3.157 (0.75–16.34)	0.116	2.852 (0.68–14.74)	0.152	NC	NC	1.238 (0.27–5.09)	0.769	1.13 (0.22–4.91)	0.875	0.777 (0.04–5.68)	0.824
**Gravidity**																		
1–3	NC	NC	0.833 (0.14–4.94)	0.834	NC	NC	1.095 (0.18–6.49)	0.916	0.456 (0.06–2.59)	0.380	0.641 (0.07–13.57)	0.719	0.875 (0.11–5.02)	0.884	1.333 (0.17–7.81)	0.76	5.00 (0.57–36.24)	0.134
4–5	0.805 (0.03–9.01)	0.862	1.093 (0.34–3.46)	0.879	0.805 (0.04–9.01)	0.862	1.467 (0.46–4.75)	0.513	1.67 (0.53–5.43)	0.381	0.794 (0.10–4.54)	0.800	1.368 (0.42–4.79)	0.608	2.933 (0.76–14.61)	0.122	1.259 (0.22–9.79)	0.801
≥6	0.685 (0.06–15.52)	0.771	0.992 (0.27–3.63)	0.989	0.685 (0.06–15.52)	0.770	0.592 (0.15–2.12)	0.422	0.812 (0.22–2.90)	0.747	1.875 (0.26–37.85)	0.562	0.731 (0.17–2.72)	0.645	0.154 (0.01–0.92)	**0.038**	NC	NC
**Parity**																		
≥2	0.088 (0.01–2.36)	0.126	NC	NC	0.088 (0.01–2.36)	0.126	NC	NC	NC	NC	0.238 (0.02–5.65)	0.313	NC	NC	NC	NC	NC	NC
3–4	2.947 (0.26–66.05)	0.374	2.182 (0.70–7.05)	0.177	2.947 (0.26–66.05)	0.374	4.091 (1.26–14.55)	**0.017**	1.179 (0.38–3.67)	0.774	1.444 (0.24–8.59)	0.673	3.967 (1.14–16.45)	0.029	2.237 (0.62–9.38)	0.223	4.167 (0.61–83.29)	0.158
≥5	1.133 (0.10–25.43)	0.921	0.777 (0.24–2.49)	0.670	1.133 (0.11–25.43)	0.921	0.388 (0.11–1.26)	0.116	1.417 (0.44–4.62)	0.555	1.143 (0.19–8.91)	0.884	0.367 (0.08–1.28)	0.120	0.628 (0.15–2.29)	0.490	0.317 (0.02–2.19)	0.267
**Menopause**	NC	NC	1.211 (0.25–5.75)	0.804	NC	NC	0.909 (0.19–4.31)	0.902	1.00 (0.21–4.74)	>0.99	1.057 (0.05–8.03)	0.962	0.926 (0.19–5.02)	0.923	0.592 (0.12–3.26)	0.523	0.315 (0.05–2.63)	0.258
**Clinical symptoms**																		
Metrorrhagia	NC	NC	5.474 (0.74–111.7)	0.100	NC	NC	4.182 (0.56–85.22)	0.172	1.568 (0.24–12.77)	0.636	NC	NC	NC	NC	0.545 (0.08–4.52)	0.541	NC	NC
Pelvic pain	NC	NC	NC	NC	NC	NC	NC	NC	0.479 (0.02–5.33)	0.547	NC	NC	NC	NC	NC	NC	NC	NC
Tumor	NC	NC	0.846 (0.03–22.22)	0.907	NC	NC	1.087 (0.04–28.54)	0.954	1.00 (0.04–26.25)	>0.99	NC	NC	NC	NC	NC	NC	NC	NC
**FIGO Staging**																		
0	0.172 (0.01–1.94)	0.149	0.8 (0.23–2.79)	0.723	0.172 (0.01–1.94)	0.149	0.497 (0.13–1.73)	0.275	0.444 (0.12–1.55)	0.205	0.333 (0.05–2.03)	0.222	0.628 (0.15–2.29)	0.490	0.6198 (0.12–2.47)	0.512	1.333 (0.17–7.81)	0.76
IA/B	0.764 (0.06–17.26)	0.834	0.136 (0.03–0.53)	**0.003**	0.764 (0.06–17.26)	0.834	0.195 (0.04–0.75)	**0.016**	0.671 (0.18–2.32)	0.528	0.75 (0.128–5.912)	0.760	0.208 (0.03–0.91)	**0.036**	0.619 (0.12–2.47)	0.512	NC	NC
IIA/B	NC	NC	2 (0.53–8.55)	0.308	NC	NC	1.729 (0.46–6.78)	0.412	1.00 (0.26–3.74)	>0.99	1.667 (0.23–33.75)	0.642	0.857 (0.19–3.26)	0.824	1.4 (0.32–5.55)	0.641	1.7 (0.21–10.14)	0.581
IIIA/B	NC	NC	NC	NC	NC	NC	NC		9.333 (1.47–182.6)	**0.015**	NC	NC	NC	NC	0.833 (0.11–4.23)	0.835	3.167 (0.38–20.51)	0.258
IVA/B	NC	NC	0.846 (0.032–22.22)	0.907	NC	NC	1.087 (0.04–28.54)	0.954	1.00 (0.04–26.25)	>0.99	NC	NC	NC	NC	NC	NC	NC	NC
**Previous HPV** **screening**	NC	NC	1.08 (0.25–4.92)	0.917	NC	NC	1.447 (0.34–6.59)	0.616	0.762 (0.16–3.27)	0.712	1.111 (0.15–22.81)	0.927	0.866 (0.16–3.81)	0.853	2.48 (0.53–11.26)	0.241	6.333 (0.98–42.27)	0.052

^a^ *p* values were calculated using Pearson χ^2^ or Fisher exact tests and significant *p* values are presented in bold. ^b^ Not converged, the logistic regression was not possible because the dependent variable had only one value giving a perfect separation with the predictor factor. ^&^ Gardasil^®^ vaccine HR-HPV (HPV-16 and HPV-18). ^£^ Gardasil-9^®^ vaccine HR-HPV (HPV-16, HPV-18, HPV-31, HPV-33, HPV-45, HPV-52, and HPV-58). Abbreviations: CI, confidence interval; cOR, crude odds ratio; FIGO: International Federation of Gynecology and Obstetrics; HPV, human papillomavirus; HR-HPV, high-risk HPV; NC, not converged.

**Table 3:** Risk factors associated with HPV outcomes using multivariate logistic regression analysis in 50 adult women living in Nouakchott, Mauritania.

	Multiple HPV		Multiple HR-HPV		^&^ Gardasil^®^-HR-HPV		Multiple ^£^ Gardasil-9^®^HR-HPV		Nonvaccine HR-HPV		Possibly Oncogenic HPV	
	aOR (95% CI)	*p* ^a^	aOR (95% CI)	*p*	aOR (95% CI)	*p*	aOR (95% CI)	*p*	aOR (95% CI)	*p*	aOR (95% CI)	*p*
**Age (years)**												
30–39	NA ^b^	NA	NA	NA	NA	NA	NA	NA	NA	NA	NA	NA
40–49	NA	NA	NA	NA	NA	NA	NA	NA	NA	NA	NA	NA
50–59	NA	NA	NA	NA	NA	NA	NA	NA	NA	NA	NA	NA
60–69	NA	NA	2.83 (0.74–10.82)	0.127	NA	NA	4.25 (1.17–15.42)	**0.028**	NA	NA	NA	NA
≥70	NA	NA	NA	NA	NA	NA	NA	NA	NA	NA	NA	NA
**Education level**												
Never	NA	NA	NA	NA	NA	NA	NA	NA	NA	NA	NA	NA
Elementary	NA	NA	NA	NA	NA	NA	NA	NA	NA	NA	NA	NA
High school	NA	NA	NA	NA	NA	NA	NA	NA	NA	NA	7.8 (1.22–49.67)	**0.03**
University	NA	NA	NA	NA	NA	NA	NA	NA	NA	NA	NA	NA
**Marital status**												
Living in couple	NA	NA	NA	NA	NA	NA	NA	NA	NA	NA	NA	NA
Divorced/Widowed	NA	NA	NA	NA	NA	NA	NA	NA	NA	NA	NA	NA
**Gravidity**												
1–3	NA	NA	NA	NA	NA	NA	NA	NA	NA	NA	NA	NA
4–5	NA	NA	NA	NA	NA	NA	NA	NA	NA	NA	NA	NA
≥6	NA	NA	NA	NA	NA	NA	NA	NA	0.15 (0.18–1.32)	0.088	NA	NA
**Parity**												
≥2	NA	NA	NA	NA	NA	NA	NA	NA	NA	NA	NA	NA
3–4	NA	NA	4.22 (1.10–16.10)	**0.035**	NA	NA	NA	NA	NA	NA	NA	NA
≥5	NA	NA	NA	NA	NA	NA	NA	NA	NA	NA	NA	NA
**Menopause**	NA	NA	NA	NA	NA	NA	NA	NA	NA	NA	NA	NA
**Clinical symptoms**												
Metrorrhagia	NA	NA	NA	NA	NA	NA	NA	NA	NA	NA	NA	NA
Pelvic pain	NA	NA	NA	NA	NA	NA	NA	NA	NA	NA	NA	NA
Tumor	NA	NA	NA	NA	NA	NA	NA	NA	NA	NA	NA	NA
**FIGO Staging**												
0	NA	NA	NA	NA	NA	NA	NA	NA	NA	NA	NA	NA
IA/B	0.136 (0.03–0.58)	**0.007**	0.16 (0.03–0.77)	**0.022**	NA	NA	0.19 (0.04–1.07)	0.06	NA	NA	NA	NA
IIA/B	NA	NA	NA	NA	NA	NA	NA	NA	NA	NA	NA	NA
IIIA/B	NA	NA	NA	NA	9.33 (1.05–82.78)	**0.045**	NA	NA	NA	NA	NA	NA
IVA/B	NA	NA	NA	NA	NA	NA	NA	NA	NA	NA	NA	NA
**Previous HPV screening**	NA	NA	NA	NA	NA	NA	NA	NA	NA	NA	NA	NA

^a^ *p* values were calculated using Pearson χ^2^ or Fisher exact tests and significant *p* values are presented in bold. ^b^ Not attributable for variables for which the cOR was not significant in univariate analyses (*p* > 0.05). ^&^ Gardasil^®^ vaccine HR-HPV (HPV-16 and HPV-18). ^£^ Gardasil-9^®^ vaccine HR-HPV (HPV-16, HPV-18, HPV-31, HPV-33, HPV-45, HPV-52, and HPV-58). Abbreviations: aOR, adjusted odds ratio; CI, confidence interval; FIGO: International Federation of Gynecology and Obstetrics; HPV, human papillomavirus; HR-HPV, high-risk HPV; NA, not attributable.

**Table 4:** Risk factors associated with high-grade cervical lesions and invasive cervical carcinoma using simple and multivariate logistic regression analysis.

	CIN2/3				SCC				ADC			
	cOR (95% CI)	*p* ^a^	aOR (95% CI)	*p*	cOR (95% CI)	*p*	aOR (95% CI)	*p*	cOR (95% CI)	*p*	aOR (95% CI)	*p*
**Age (years)**												
30–39	5.88 (0.52–132.3)	0.149	NA ^c^	NA	0.26 (0.01–2.88)	0.265	NA	NA	NC ^b^	NC	NA	NA
40–49	2.48 (0.53–11.26)	0.241	NA	NA	0.37 (0.08–1.62)	0.185	NA	NA	1.58 (0.07–14.33)	0.715	NA	NA
50–59	1.94 (0.48–7.48)	0.338	NA	NA	0.86 (0.24–3.37)	0.830	NA	NA	NC	NC	NA	NA
60–69	0.166 (0.03–0.72)	**0.015**	0.32 (0.05–1.89)	0.209	2.29 (0.68–8.55)	0.181	NA	NA	5.12 (0.60–108.1)	0.138	NA	NA
≥70	0.62 (0.03–4.67)	0.665	NA	NA	2.43 (0.32–49.61)	0.412	NA	NA	NC	NC	NA	NA
**Education level**												
Never schooled	0.49 (0.13–1.73)	0.275	NA	NA	2.57 (0.79–9.03)	0.116	NA	NA	0.33 (0.02–2.82)	0.325	NA	NA
Elementary school	0.29 (0.04–1.31)	0.113	NA	NA	1.18 (0.34–4.49)	0.796	NA	NA	8.5 (0.98–181.2)	0.05	NA	NA
High school	6.11 (1.26–34.78)	**0.024**	1.63 (0.19–13.62)	0.651	0.27 (0.04–1.26)	0.095	NA	NA	NC	NC	NA	NA
University	5.83 (0.52–132.3)	0.149	NA	NA	0.26 (0.01–2.88)	0.265	NA	NA	NC	NC	NA	NA
**Marital status**												
Living in couple/married	4.33 (0.70–84.22)	0.125	NA	NA	0.71 (0.14–3.01)	0.655	NA	NA	0.21 (0.02–1.96)	0.158	NA	NA
Divorced/Widowed	0.23 (0.02–1.42)	0.125	NA	NA	1.4 (0.33–7.25)	0.655	NA	NA	4.75 (0.51–44.78)	0.158	NA	NA
**Gravidity**												
1–3	6.8 (1.15–54.58)	**0.034**	2.35 (0.28–19.08)	0.425	0.23 (0.03–1.34)	0.102	NA	NA	NC	NC	NA	NA
4–5	0.5 (0.14–1.77)	0.279	NA	NA	1.52 (0.46–5.03)	0.482	NA	NA	1.93 (0.22–40.66)	0.565	NA	NA
≥6	0.71 (0.14–2.87)	0.641	NA	NA	1.37 (0.36–5.82)	0.645	NA	NA	0.94 (0.04–8.21)	0.961	NA	NA
**Parity**												
≥2	5.83 (0.52–132.3)	0.149	NA	NA	0.25 (0.01–2.88)	0.265	NA	NA	NC	NC	NA	NA
3–4	0.42 (0.12–1.47)	0.177	NA	NA	2.38 (0.74–8.02)	0.1459	NA	NA	0.71 (0.07–6.28)	0.736	NA	NA
≥5	1.5 (0.41–5.34)	0.532	NA	NA	0.56 (0.16–1.88)	0.353	NA	NA	1.87 (0.2–16.85)	0.551	NA	NA
**Menopause**	0.31 (0.06–1.53)	0.147	NA	NA	3.72 (0.79–20.42)	0.095	NA	NA	0.54 (0.05–11.74)	0.628	NA	NA
**Clinical symptoms**												
Metrorrhagia	0.22 (0.02–1.45)	0.113	NA	NA	3 (0.45–24.68)	0.249	NA	NA	NC	NC	NA	NA
Pelvic pain	NC	NC	NA	NA	NC	NC	NA	NA	NC	NC	NA	NA
Tumor	NC	NC	NA	NA	NC	NC	NA	NA	NC	NC	NA	NA
**FIGO staging**												
0	NC	NC	NA	NA	NC	NC	NA	NA	NC	NC	NA	NA
IA/B	NC	NC	NA	NA	2.62 (0.67–13.07)	0.169	NA	NA	9.54 (1.09–204.2)	**0.041**	9.54 (0.89–101.33)	0.061
IIA/B	NC	NC	NA	NA	8.91 (1.50–171.0)	**0.013**	11.12 (1.12–109.99)	**0.039**	1.10 (0.05–9.28)	0.961	NA	NA
IIIA/B	NC	NC	NA	NA	NC	NC	NA	NA	NC	NC	NA	NA
IVA/B	NC	NC	NA	NA	NC	NC	NA	NA	NC	NC	NA	NA
**Previous HPV screening**	8.25 (1.79–46.46)	**0.006**	2.97 (0.36–24.32)	0.31	0.21 (0.04–0.92)	**0.037**	0.16 (0.03–0.94)	**0.043**	NC	NC	NA	NA
**HPV genotypes distribution profile**												
HPV	0.17 (0.01–1.94)	0.149	NA	NA	3.87 (0.34–87.11)	0.265	NA	NA	NC	NC	NA	NA
Multiple HPV	0.8 (0.23–2.79)	0.724	NA	NA	0.91 (0.27–2.89)	0.868	NA	NA	2.75 (0.32–57.87)	0.367	NA	NA
HR-HPV	0.17 (0.01–1.94)	0.149	NA	NA	3.87 (0.34–87.11)	0.265	NA	NA	NC	NC	NA	NA
Multiple HR-HPV	0.49 (0.13–1.73)	0.275	NA	NA	1.25 (0.39–4.07)	0.705	NA	NA	3.57 (0.42–75.19)	0.251	NA	NA
Possibly oncogenic HPV	1.33 (0.17–7.81)	0.76	NA	NA	1.143 (0.19–8.91)	0.884	NA	NA	NC	NC	NA	NA
^&^ Gardasil^®^ HR-HPV	0.44 (0.12–1.55)	0.205	NA	NA	1 (0.31–3.21)	>0.999	NA	NA	NC	NC	NA	NA
^£^ Gardasil-9^®^ HR-HPV	0.33 (0.05–2.03)	0.222	NA	NA	1.93 (0.32–11.60)	0.454	NA	NA	NC	NC	NA	NA
Multiple Gardasil-9^®^ HR-HPV	0.63 (0.15–2.29)	0.490	NA	NA	1.2 (0.36–4.22)	0.767	NA	NA	1.87 (0.21–16.85)	0.550	NA	NA
Nonvaccine HR-HPV	0.62 (0.12–2.47)	0.511	NA	NA	1.59 (0.43–6.70)	0.490	NA	NA	0.84 (0.04–7.31)	0.888	NA	NA

^a^ *p* values were calculated using Pearson χ^2^ or Fisher exact tests and significant *p* values are presented in bold. ^b^ Not converged, the logistic regression was not possible because the dependent variable had only one value giving a perfect separation with the predictor factor. ^c^ Not attributable for variables for which the cOR was not significant in univariate analyses (*p* > 0.05). ^&^ Gardasil^®^ vaccine HR-HPV (HPV-16 and HPV-18). ^£^ Gardasil-9^®^ vaccine HR-HPV (HPV-16, HPV-18, HPV-31, HPV-33, HPV-45, HPV-52, and HPV-58). Abbreviations: aOR, adjusted odds ratio; CI, confidence interval; cOR, crude odds ratio; FIGO: International Federation of Gynecology and Obstetrics; HPV, human papillomavirus; HR-HPV, high-risk HPV; NA, not attributable; NC, not converged.

## Data Availability

The data that support the conclusions of this study are available from the corresponding author upon reasonable request.
